# Prenatal Diagnosis and Management of a Rare Central Tendon Defect Type of Congenital Diaphragmatic Hernia with a Massive Pericardial Effusion

**DOI:** 10.1155/2020/6798253

**Published:** 2020-01-22

**Authors:** Inas Babic, Haifa Al-Jobair, Osama Al Towaijri, Huda Al-Shammary, Merna Atiyah, Jamal Al Hudhaif, Amer Ammari

**Affiliations:** ^1^Division of Maternal Fetal Medicine, Department of Obstetrics & Gynecology, Prince Sultan Military Medical City, Riyadh, Saudi Arabia; ^2^Department of Pediatric Cardiology, Prince Sultan Cardiac Center, Riyadh, Saudi Arabia; ^3^Department of Pediatric Surgery, Prince Sultan Military Medical City, Riyadh, Saudi Arabia; ^4^Division of Neonatal Medicine, Department of Pediatrics, Prince Sultan Military Medical City, Riyadh, Saudi Arabia

## Abstract

The central tendon defect type of congenital diaphragmatic hernia (CDH) is extremely rare and usually associated with a significant pericardial effusion. Prenatal diagnostic ultrasound features of this quite rare entity remain often overlooked or misdiagnosed. There is a dearth of literature about the role of prenatal intervention, often through an elective pericardiocentesis, for the prevention of lung hypoplasia and to decrease the overall neonatal morbidity and mortality. To the best of our knowledge, till date, there is only one case that was subjected to a prenatal intervention. Here, we present a second case of a central tendon defect type of CDH with a large pericardial effusion that was subjected to a prenatal transthoracic pericardiocentesis. Although smooth intubation and ventilation were performed immediately after birth, the infant suffered for several months from respiratory instability. Laparoscopic central tendon hernia repair was performed, and neonate was discharged home at seven months of age. Although prenatal pericardiocentesis may facilitate smoother postnatal intubation and ventilation, its broader effect on respiratory function is uncertain and still remains elusive.

## 1. Introduction

Congenital diaphragmatic hernia (CDH) is a rare, major structural abnormality with a high mortality rate [1]. It is a defect of the diaphragm, which often leads to a herniation of the abdominal organs into the thoracic cavity. It has been traditionally classified according to its anatomic location into two major groups: most common, posterolateral (Bochdalek hernia) and rarer, anterior (hernia of Morgagni). However, it has been found that these two entities carry considerable imprecision as neither all defects are always localized strictly anteriorly or posteriorly nor it always demonstrates a single defect, but rather occasionally as a combination of coexisting two or more distinct diaphragmatic defects [2, 3]. Therefore, anterior hernias have been further categorized into three subtypes: (1) retrosternal or parasternal hernias (Morgagni or Morgagni-Larrey hernias), the most anterior defect of the diaphragm; (2) anterior hernias, which are anteriorly localized with an extension into the anterior central tendon, due to the defect in septum transversum; and (3) central hernias, which are categorized by the defects in the central tendinous portion of the diaphragm [4]. CDH of central tendon defect is uncommon and is often associated with a large pericardial effusion and lung hypoplasia [5, 6]. Treatment is usually surgical in neonatal or early infancy period. Respiratory failure due to lung hypoplasia is the leading cause for an early death in affected neonates. Whether early prenatal management should regularly include pericardiocentesis for lung expansion and ultimately prevention of lung hypoplasia or to be deferred into postnatal period is not clearly defined in the medical literature. There are very few case reports that speculated about this issue [7, 8]. Here, we report our experience of prenatal and postnatal management of a rare case of central tendon defect of CDH with a large pericardial effusion.

## 2. Case Presentation

A 21-year-old Middle Eastern woman, primigravida, was referred due to a suspected abnormality found in the fetal chest on the antenatal ultrasound. She was otherwise a healthy woman without any significant pregnancy-related medical issues. She and her husband are not consanguineous. No significant family history on their both sides.

**Figure 1:**
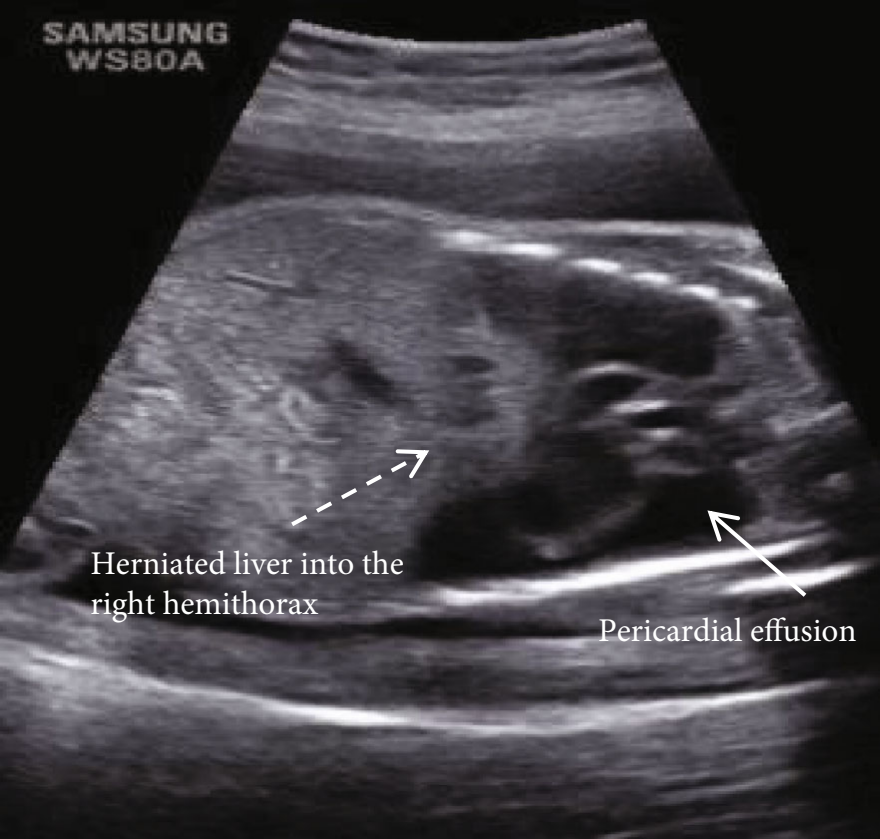
Prenatal ultrasound image of coronal section of the fetal thorax and abdomen, showing a large pericardial collection (full arrow), and an eventrated diaphragm with part of the liver is in the thoracic cage towards the right (interrupted arrow).

**Figure 2:**
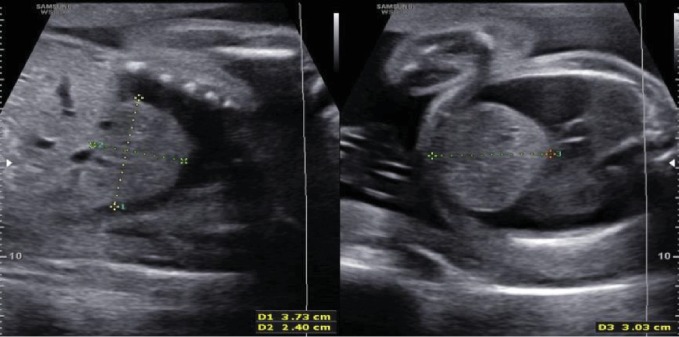
Prenatal ultrasound images of sagittal and transverse sections of fetal thorax, showing large liver lobe protruding into the thoracic cavity.

The ultrasound at 28^th^ week of gestation revealed a large pericardial effusion and compressed lungs, posteriorly against the chest wall. There was diaphragmatic eventration on the right side with a solid mass protruding into the right hemithorax with significant lung compression (Figure 1). The mass was the left lobe of the liver, measuring 3.73 cm × 2.40 cm × 3.03 cm, not compressing the heart (Figure 2). Fetal echocardiogram showed normal heart structure with global massive pericardial effusion, compressing lungs bilaterally with good biventricular function. The cardiac magnetic resonance imaging (cardiac MRI) showed no systemic vascular compression from the solid mass. There were no signs of cardiac tamponade.

Parental multidisciplinary counseling was carried out by a team consisted of maternal-fetal medicine, neonatology, cardiology, and pediatric surgery experts and decided for conservative treatment without intervention until term. The woman received two doses of corticosteroids to enhance lung maturity; however, she declined prenatal genetic testing for karyotype and microarray comparative genomic hybridization (array CGH). Due to persistent massive pericardial effusion and concerning issues of a likely need for an immediate neonatal pericardiocentesis to facilitate ventilation and lung expansion, the woman agreed to undergo transthoracic fetal pericardiocentesis at 37th week of gestation. Twenty-seven milliliter of serosanguinous fluid was aspirated from the pericardial space under ultrasonographic guidance. The baby had fetal bradycardia, and a cesarean section was performed.

**Figure 3:**
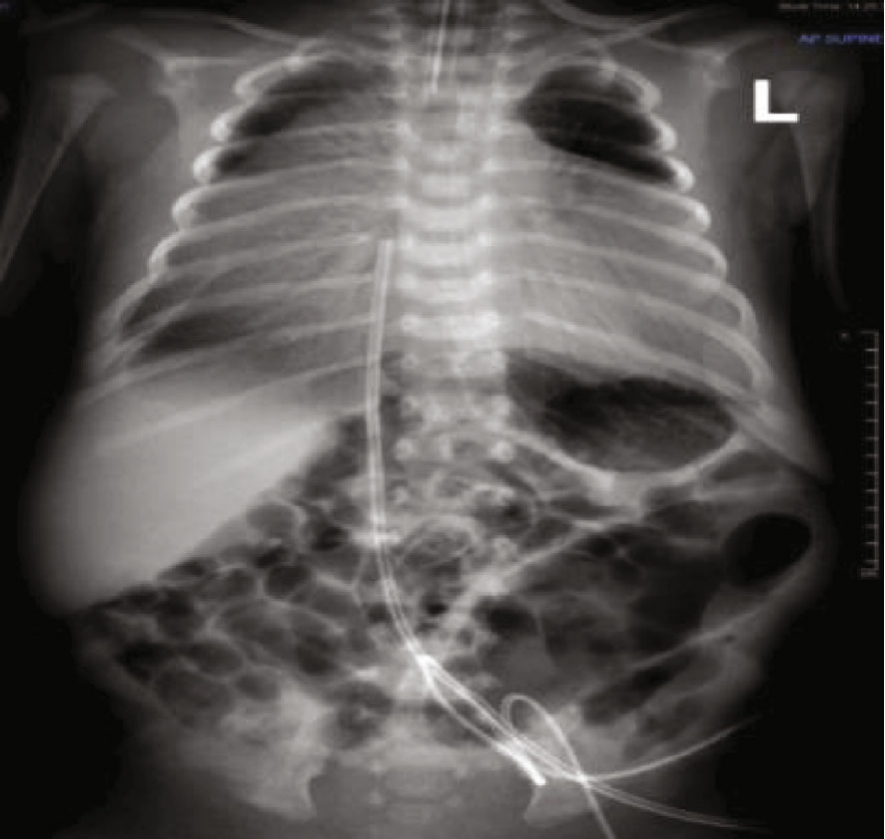
The initial chest and abdominal radiograph, showing a small lung volume, a large cardiothymic shadow, and air bronchogram streaks indicative of neonatal surfactant deficiency syndrome.

**Figure 4:**
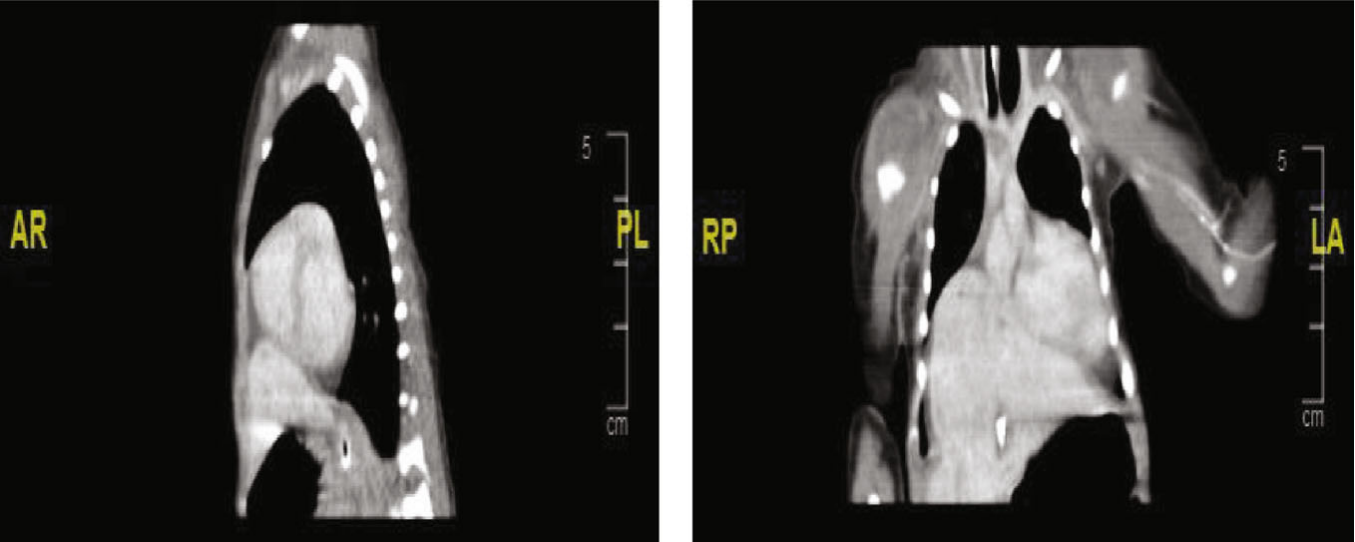
Preoperative computed tomogram cuts, showing the herniated liver into the thoracic cavity, large pericardial effusion, and compressed lungs.

The outcome was a male baby with a birth weight of 2330 grams (5^th^ centile as per neonatal male growth chart). The fetus was small for the gestational age. The APGAR score was 6 and 8 at 1^st^ and 5^th^ minute, respectively. The neonate was electively intubated and admitted to the neonatal intensive care unit. The chest radiograph (Figure 3) showed dense haziness of both lungs obliterating the normal cardiac shadow, air bronchograms, and features of compressed lungs. High inflation pressures were required to maintain preductal saturation in the normal range. There was no need for postnatal pericardial fluid drainage. The transthoracic echocardiogram showed a “large” patent ductus arteriosus (PDA) with left-to-right shunting and a rim of pericardial effusion. The ductus arteriosus became “tiny” in size on subsequent echocardiograms. The chest computed tomogram with contrast suggested an eventration of the right hemidiaphragm with the left lobe of the liver residing in the chest. Small pericardial and pleural effusions were also seen (Figure 4). The neonate was taken to surgery, and during the laparoscopic repair of the hernia, a large infracardiac central tendon diaphragmatic defect was seen and repaired without the need for a synthetic mesh.

Postoperative tomogram of the chest evidenced an elevation of the left hepatic lobe and the medial aspect of the right lobe into the central part of the hemithorax in the retrosternal region, with a mass effect displacing the heart superiorly, posteriorly and to the left side. The herniated liver tissue was smaller than the preoperative study.

The baby was successfully extubated and tolerated breathing room air, only to require continuous positive airway pressure (CPAP) and oxygen after an inguinal hernia repair at the age of six months. At the age of seven months, he was breathing room air and was discharged in a stable condition.

## 3. Discussion

We presented an uncommon, interesting case of a central tendon defect type of CDH. This is the second reported case that describes prenatal intervention through an ultrasound-guided transthoracic pericardiocentesis. Central tendon defect of CDH is a very rare entity [9]. Developmental defect in the retrosternal portion of septum transversum contributes to a herniation of different abdominal organs into the thoracic cavity at different times during the gestation. Earlier prenatal recognition may accord well with parental counseling and optimization of various management options. Unfortunately, quite often, cases are diagnosed postnatally, at infancy, or even later during childhood. There are seventeen cases of central CDH reported to date. Still, only six cases have been diagnosed prenatally by ultrasound at different gestational ages [10–13]. One of them underwent elective termination of pregnancy at eighteen weeks after parental counseling, and five had closure by the repair during early neonatal period with overall good outcomes.

Surgical interventions were mainly represented with an open approach; yet, there are only two cases that underwent transabdominal and transthoracic laparoscopic repairs by using a patch. The outcomes were overall satisfactory, and both babies did well postoperatively [6, 14]. Ours is the third to endure a laparoscopic approach but without need for a patch, rather just suture closure of the defect.

Surprisingly, the two neonates had an absence of respiratory distress despite massive pericardial effusion and compressed lungs but required pericardiocentesis prior to an elective surgery [12, 13]. One baby required only short-term respiratory support following respiratory recovery within 2 days [8]. Three prenatally diagnosed cases including ours developed dyspnea and variable ranges of respiratory distress requiring ventilatory support [10, 14].

Pericardial effusion enormously contributes to lung hypoplasia, respiratory distress, and high frequency oxygen support. The etiology for pericardial effusion evolution is not definitely understood, but yet, there are three possible hypotheses: accumulation of lymph due to thoracic duct compression, congestion, and transudation due to venous obstruction or due to mechanical irritation from the herniated viscera [8, 15]. Idea of prenatal intervention in a view of pericardial fluid aspiration and lung expansion has been discussed in a scanty literature. Fetal pericardiocentesis is aimed at (1) decompressing the fetal chest to allow the lungs to expand, thus avoiding respiratory morbidity and fetal demise, (2) theoretically preventing hydrops fetalis by improving venous drainage, and (3) preventing or treating cardiac tamponade that may manifest after delivery [16].

Up to our knowledge, there had been only two cases of prenatal pericardiocentesis in fetuses with broad spectrum CDH [10, 17]. Antiñolo et al. reported Morgagni's CDH and speculated that prenatal pericardiocentesis likely prevented severe lung hypoplasia at early gestation (21 and 22 weeks of gestation), which facilitated good neonatal outcome [17]. On the contrary, Taketani et al., in their report of a case with intrapericardial CDH, had diagnosed severe lung hypoplasia with persistent pulmonary hypertension in the newborn, despite draining all pericardial fluids at 27 weeks of gestation that led to obvious lung expansion [10]. In one case, the author combined pericardiocentesis with peri-cardio-amniotic shunting in a fetus with severe pericardial effusion ensuing intracardiac teratoma associated with hydrops fetalis [18]. The outcome was unfortunate, ended with intrauterine fetal death as early as 24 weeks of gestation.

We describe here, the second case of prenatal transthoracic pericardiocentesis in a fetus with a rare central tendon defect type of CDH. Although the procedure went on smoothly, without concerning issues rising up during or immediately post procedure, the fetus did develop unrecovered bradycardia which necessitated immediate delivery. The effusion was almost completely resolved. The postnatal echocardiogram showed only a thin rim of fluid in the pericardial space. No major intracardiac anomalies were detected.

Altogether, it is challenging to draw a firm cause-effect association with recommendation of strong certainty. However, beyond any doubt, it is absolved that prolonged lung compression, leading to lung hypoplasia is the major factor causing neonatal morbidity and mortality. Due to paucity of the literature, we can only speculate that prenatal intervention may or may not modify the clinical outcome. On the one hand, prenatal intervention may have contributed to more effective ventilation and overall respiratory care postnatally, as noticed in our case. On the other hand, it showed limited effect on the respiratory function postnatally.

Perhaps, if pericardial fluid aspiration was done earlier (for example, at 27 weeks as firstly presented in our case), the respiratory function might have been improved with decreased ventilation period and faster infant's respiratory recovery. More cases are required to be reported, for the purpose of reaching more certain conclusion.
